# Bedside Assessment of PEEP-Induced Volume and Mechanical Power in Mechanically Ventilated Adults Without ARDS: A Post Hoc Analysis

**DOI:** 10.3390/medsci14040451

**Published:** 2026-08-01

**Authors:** Adrián Gallardo, Armando Díaz-Cabrera, Cristian Deana, Luigi Vetrugno, Mauro Castro-Sayat

**Affiliations:** 1 Servicio de Kinesiología y Cuidados Respiratorios, Sanatorio Clínica Modelo de Morón, Morón C1015, Argentina; adriankgallardo@gmail.com; 2 Departamento de Ciencias de la Salud, Kinesiología y Fisiatría, Universidad Nacional de la Matanza, San Justo B1754, Argentina; 3 WeVent, Grupo Internacional de Ventilación Mecánica, Viña del Mar 2520000, Chile; 4 Hospital San Juan de Dios, Santiago 8350488, Chile; armandoadc@gmail.com; 5 Exercise and Rehabilitation Sciences Institute, School of Physical Therapy, Faculty of Rehabilitation Sciences, Universidad Andres Bello, Santiago 7591538, Chile; 6 Anesthesia and Intensive Care 1, Department of Anesthesia and Intensive Care, Health Integrated Agency of Friuli Centrale, 33100 Udine, Italy; 7 Anesthesia and Intensive Care Unit, Department of Emergency, Health Integrated Agency of Friuli Centrale, Tolmezzo Hospital, 33028 Tolmezzo, Italy; luigi.vetrugno@asufc.sanita.fvg.it; 8 Departamento de Emergencias, Unidad de Soporte Respiratorio No Invasivo, Hospital Juan A Fernández, Buenos Aires C1425, Argentina; maurocastrosayat@gmail.com

**Keywords:** mechanical ventilation, mechanical power, lung volume, positive end expiratory pressure, alveolar recruitment

## Abstract

Background/Objectives: Positive end-expiratory pressure (PEEP) increases end-expiratory lung volume through the addition of PEEP-induced lung volume (PEEPVol), potentially affecting respiratory mechanics and energy load. However, its physiological impact in patients without lung injury remains poorly characterized. We hypothesized that PEEPVol would be closely associated with respiratory system loading beyond conventional respiratory mechanics variables. Methods: We conducted a secondary analysis of a prospective physiological study including 16 deeply sedated, mechanically ventilated adults without lung disease. A standardized incremental PEEP titration (0–16 cmH_2_O, steps of 4 cmH_2_O) was performed under volume-controlled ventilation. At each step, respiratory mechanics were assessed, including static compliance (Cstat), driving pressure, plateau pressure, and mechanical power. PEEPVol was estimated as PEEP × Cstat, and strain indices were derived relative to predicted functional residual capacity. Linear mixed-effects models evaluated changes across PEEP levels, and associations were initially explored using Spearman correlation and linear regression. To account for repeated measurements within subjects, confirmatory linear mixed-effects regression models were subsequently performed using patients as a random intercept. Results: Incremental PEEP significantly increased PEEPVol, plateau pressure, mechanical power, and both static and global strain (all *p* < 0.001), while changes in compliance and driving pressure were minimal and not clinically meaningful. PEEPVol showed moderate-to-strong correlations with plateau pressure (ρ = 0.68) and mechanical power (ρ = 0.76) and explained 43% and 56% of their variance, respectively (all *p* < 0.001);these associations remained significant in additional linear mixed-effects regression analyses accounting for within-subject correlation. PEEPVol also correlated strongly with static strain (ρ = 0.93) and global strain (ρ = 0.83); however, because static strain is calculated as PEEPVol/FRCt, this association is mathematically expected rather than an independent physiological finding and is reported here only for completeness. Notably, 11.1% of measurements exceeded Pplat > 30 cmH_2_O and 61.9% exceeded MP ≥ 17 J/min, even at moderate PEEP levels. Conclusions: In patients without lung injury, PEEP-induced increases in lung volume are strongly associated with higher mechanical load and strain, despite minimal changes in compliance or driving pressure. PEEPVol may represent a promising physiological surrogate of static lung deformation and energy transfer, whose potential to improve bedside detection of occult overdistension warrants validation against direct imaging or physiological measurements in larger prospective studies.

## 1. Introduction

Mechanical ventilation (MV) remains the cornerstone of life-support in the intensive care unit (ICU), serving as the primary intervention for acute respiratory failure. The therapeutic objectives of MV extend beyond ensuring adequate gas exchange; they encompass the mitigation of work of breathing and the prevention of ventilator-induced lung injury (VILI) and ventilator-induced diaphragmatic dysfunction (VIDD) to facilitate respiratory recovery.

Current protective strategies include low tidal volume ventilation (~6–8 mL/kg of predicted body weight) [1], the limitation of driving pressure [2], individualized positive end-expiratory pressure (PEEP) titration [3], and the minimization of mechanical power [4]. While these approaches effectively mitigate pulmonary stress and strain, their downstream effects on diaphragmatic mechanics are increasingly recognized as clinically significant [5]. Excessive lung volumes and elevated transpulmonary pressures induce alveolar overdistension, generating substantial stress and strain within the lung parenchyma [6,7].

Conversely, clinical factors frequently encountered during MV—such as the administration of sedatives and analgesics, supine positioning, and elevated intra-abdominal pressure—consistently reduce functional residual capacity (FRC). This reduction in end-expiratory lung volume could precipitate atelectasis and impaired gas exchange. Within this framework of lung–diaphragm interaction, PEEP is applied to restore end-expiratory lung volume through the addition of a gas volume, termed the “PEEP volume” (PEEPVol). While PEEP may enhance lung recruitment and oxygenation, the resulting increase in lung volume directly modulates diaphragmatic position, respiratory system mechanics, and loading conditions. Notably, PEEPVol could be governed by the respiratory system’s elastance, which varies substantially among individuals. Consequently, PEEP optimization remains a central clinical controversy: higher PEEP levels may prevent alveolar collapse and stabilize lung units [8]; conversely, excessive PEEP may exacerbate lung overdistension, amplify mechanical power, and impose unfavorable loads on the diaphragm, potentially worsening VIDD and compromising hemodynamic stability [9]. Thus, PEEP represents a critical nexus where lung protection and diaphragm preservation may either converge or conflict.

Experimental evidence suggests that static pulmonary stress may be less injurious than dynamic stress [6,7]; however, in the absence of recruitable lung units, the accumulation of static strain via PEEPVol may unnecessarily increase the total energy load (mechanical power) delivered to the parenchyma. Although the “open lung” concept, popularized in the 1990s [8], successfully emphasized lung protection, it largely failed to account for the mechanical burden imposed on the diaphragm. In contrast, lower PEEP levels may improve diaphragmatic and lung mechanics in specific clinical settings, albeit potentially at the cost of lung derecruitment [9]. Despite these trade-offs, the role of PEEP in modulating mechanical power remains incompletely understood, as it is often treated as a static variable independent of ventilatory dynamics [10]. It is important to emphasize that achieving and maintaining a specific PEEP level requires energy expenditure, induces lung deformation, and contributes to the overall mechanical power delivered to the respiratory system, thereby increasing mechanical load and deformation patterns that have been associated with VILI in prior experimental studies [11,12]. Given the intimate mechanical coupling between the lung and diaphragm, these pathophysiological effects are likely to extend well beyond the lung parenchyma.

Based on these considerations, we hypothesized that the magnitude of PEEPVol may reflect the balance between end-expiratory lung volume expansion and the associated increase in respiratory system stress and energy transfer. Therefore, the aim of this study was to investigate, in a population of healthy lungs (without known lung disease), the relationship between incremental PEEP application, the resulting PEEPVol, and established respiratory mechanics parameters.

## 2. Materials and Methods

### 2.1. Study Design and Setting

This study is a secondary (post hoc) analysis of data derived from a prospective physiological study originally designed to evaluate the impact of an ascending positive end-expiratory pressure titration strategy on respiratory mechanics and mechanical power in patients without lung injury [13]. That primary report described how Cstat, Pplat, driving pressure, and mechanical power changed across the PEEP titration protocol but did not estimate PEEP-induced lung volume (PEEPVol) or its relationship with strain indices. The present analysis addresses a distinct research question, namely whether a bedside-derived estimate of PEEPVol adds physiological information about mechanical loading beyond the pressure-based variables already reported and therefore uses variables and analyses that were not part of the primary publication. The primary study enrolled adult patients without clinically evident pulmonary disease who required invasive mechanical ventilation under deep sedation (Richmond Agitation–Sedation Scale [RASS] score of −5) within the first 48 h following endotracheal intubation. Deep sedation ensured the absence of spontaneous respiratory effort, allowing assessment of passive respiratory mechanics.

Patients were included if no prior history of chronic lung disease was documented and no clinical, radiological, or gas exchange criteria suggestive of acute respiratory distress syndrome or other pulmonary pathology were present at the time of data collection. The underlying indications for mechanical ventilation were non-pulmonary.

The 48 h time frame was selected to minimize the influence of secondary ventilator-induced or inflammatory lung changes and to approximate baseline respiratory mechanics under controlled conditions. The study protocol was approved by the Institutional Review Board of Sanatorio Clínica Modelo de Morón (Approval No. 02/2024). The requirement for informed consent was waived due to the retrospective and observational nature of the study, which involved only anonymized data from clinical records with no intervention or additional procedures for the patients. The investigation was conducted in accordance with the ethical principles of the Declaration of Helsinki.

### 2.2. Ventilation Protocol and Data Collection

Patients underwent a standardized incremental PEEP titration protocol. PEEP levels were increased in steps of 4 cmH_2_O up to a maximum of 16 cmH_2_O, starting from zero end-expiratory pressure (ZEEP). Each level was maintained for 10 min to ensure the stabilization of respiratory mechanics. Respiratory system static compliance, plateau pressure (Pplat), DP (defined as Pplat − total PEEP), and MP were assessed at the end of each step. Ventilatory management was standardized by adjusting the respiratory rate to ensure normocapnia, with a PaCO_2_ target of 35–45 mmHg. Likewise, the minimum FiO_2_ necessary to guarantee an oxygen saturation (SpO_2_) of no less than 94% was applied. During data collection, subjects remained in the supine position with the head of the bed elevated to 45°. At each stage of PEEP titration, plateau pressure, peak pressure, and driving pressure (DP) were recorded using a 3 s inspiratory pause maneuver. Finally, the respiratory system compliance was determined through the TV/DP ratio.

Out of the 64 theoretical data points expected from the titration protocol (16 patients across 4 PEEP levels), a total of 63 measurements were ultimately analyzed (*n* = 63). This minor discrepancy was secondary to a pre-established clinical safety constraint in a single subject. During the 12 cmH_2_O PEEP step, this patient reached an upper-threshold driving pressure of 15 cmH_2_O. In strict adherence to lung-protective guidelines and to mitigate the risk of ventilator-induced lung injury, further PEEP increment to 16 cmH_2_O was suspended for this individual, resulting in one missing data point at the maximum titration step.

Mechanical ventilation was delivered via a Puritan-Bennett 840 ventilator (Medtronic, Boulder, CO, USA) in volume-controlled continuous mandatory ventilation (VC-CMV) mode. The tidal volume (TV) was set between 6 and 8 mL/kg of predicted body weight and was maintained during the whole protocol. During the procedure, monitoring was performed to ensure proper oxygenation and hemodynamic status. FiO_2_ was not required to be adjusted during the protocol.

### 2.3. Respiratory Mechanics and Derived Variables

At each PEEP level, respiratory system mechanics were assessed following a 3 s end-inspiratory occlusion under conditions of controlled mechanical ventilation and absence of spontaneous respiratory effort, ensuring quasi-static conditions. Measured parameters included static compliance (Cstat), plateau pressure (Pplat), and driving pressure (ΔP), calculated as the difference between Pplat and total PEEP.

Theoretical functional residual capacity (FRCt) was estimated using validated predictive equations based on sex, height, and age [14,15]. Although these equations provide a standardized approximation of baseline lung volume, they do not account for individual variations related to sedation, positioning, or applied PEEP:

- Males: FRCt = 2.34 × height (m) + 0.022 × age (years) − 1.23
- Females: FRCt = 2.24 × height (m) + 0.001 × age (years) − 1.00

PEEP-induced lung volume (PEEPVol) was estimated as the product of applied PEEP and static compliance (PEEP × Cstat). This approach assumes linear behavior of the respiratory system within the studied range and should be interpreted as a surrogate of volume change rather than a direct measurement.

Static and global strain were defined as PEEPVol/FRCt and (PEEPVol + TV)/FRCt, respectively. Given that strain indices are mathematically derived from PEEPVol, analyses involving these variables were interpreted with caution due to potential structural coupling.

Mechanical power (MP) was estimated using a simplified equation [4]: MP = 0.098 × respiratory rate × TV × (Ppeak − ½ΔP). This formula provides an approximation of the energy transferred to the respiratory system per unit time but does not account for the full resistive and viscoelastic components of ventilation.

### 2.4. Statistical Analysis

Descriptive statistics were used to summarize patient characteristics and respiratory variables. Categorical variables are presented as absolute counts and percentages (*n*, %), while continuous variables are expressed as mean ± standard deviation or median with interquartile range (IQR, 25th–75th percentile), according to data distribution. Normality was assessed using the Shapiro–Wilk test.

Changes in respiratory mechanics across PEEP levels (Cstat, ΔP, Pplat, PEEPVol, mechanical power, static strain, and global strain) were analyzed using linear mixed-effects models fitted by restricted maximum likelihood (REML), accounting for repeated measurements within subjects. PEEP level was included as a fixed effect and subject as a random intercept. Post hoc pairwise comparisons between PEEP levels were performed using Bonferroni adjustment.

Associations between PEEPVol and respiratory variables were initially explored using Spearman’s rank correlation coefficient (ρ). Univariable linear regression analyses were subsequently performed for variables demonstrating significant correlations. Because repeated observations obtained at different PEEP levels within the same patient are not statistically independent, these correlation and regression analyses were considered exploratory and intended primarily to illustrate the direction and magnitude of the observed associations.

To account for the repeated-measures structure of the dataset, additional linear mixed-effects regression models were performed for plateau pressure, mechanical power, static strain, and global strain. In each model, PEEP-induced lung volume (PEEPVol) was included as a fixed effect and subject as a random intercept. Regression coefficients (β), 95% confidence intervals (CI), and *p*-values were reported. These additional analyses were used to evaluate whether the observed associations remained significant after accounting for within-subject correlation.

Model assumptions were assessed graphically by visual inspection of residual-versus-fitted and normal Q–Q plots. Correlation coefficients (ρ), regression coefficients (β), coefficients of determination (r^2^), 95% confidence intervals, and *p*-values are reported where appropriate.

Analyses of changes across PEEP levels, correlation analyses, and simple linear regressions were performed using GraphPad Prism version 9 (GraphPad Software, San Diego, CA, USA). Linear mixed-effects regression analyses were performed in R (R Foundation for Statistical Computing, Vienna, Austria) using the lme4, lmerTest, emmeans, performance, and broom.mixed packages. Statistical significance was defined as a two-tailed *p*-value < 0.05.

## 3. Results

### 3.1. Study Population and Baseline Characteristics

Baseline characteristics of the 16 enrolled participants are summarized in Table 1. The cohort was predominantly male (81.3%), with a mean age of 57 ± 16 years. Estimated mean theoretical functional residual capacity (FRCt) was 3478 ± 589 mL. Patients were managed with a standardized lung-protective ventilation strategy, with a mean tidal volume of 492.5 ± 50.4 mL, corresponding to 7.27 ± 0.6 mL/kg of predicted body weight.

### 3.2. Impact of Incremental PEEP on Respiratory Mechanics

**Figure 1:**
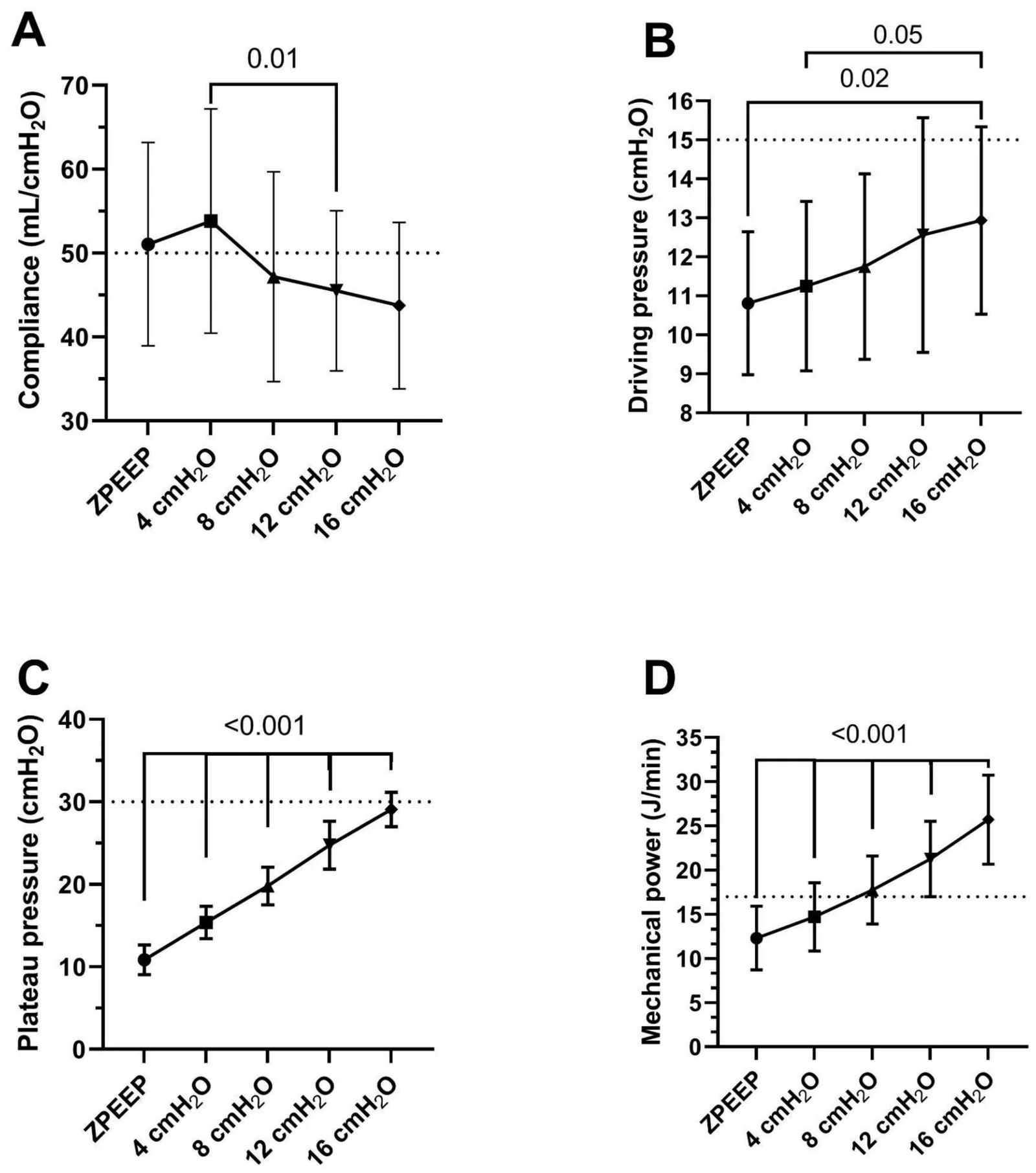
Respiratory mechanics during incremental PEEP. Data are presented as mean ± standard deviation for each PEEP level. (**A**): Static compliance (horizontal dashed line indicates a cutoff of 50 mL/cmH_2_O). (**B**): Driving pressure (horizontal dashed line indicates a cutoff of 15 cmH_2_O). (**C**): Plateau pressure (horizontal dashed line indicates a cutoff of 30 cmH_2_O). (**D**): Mechanical power (horizontal dashed line indicates a cutoff of 17 J/min). One-way ANOVA with Bonferroni post hoc comparisons was used to assess differences between PEEP levels. Statistical significance was defined as *p* < 0.05.

Incremental PEEP titration was associated with significant changes in respiratory mechanics (Figure 1). Using a linear mixed-effects model, static compliance (Cstat) showed a modest but statistically significant variation across PEEP levels (*p* = 0.008, F = 5.46). Driving pressure (ΔP) also varied significantly (*p* < 0.001, F = 9.84), although post hoc comparisons revealed that meaningful differences were mainly observed at higher PEEP levels, with ΔP increasing from ZEEP to 16 cmH_2_O by −2.12 cmH_2_O (95% CI −3.49 to −0.75; adjusted *p* = 0.002).

In contrast, plateau pressure (Pplat) and mechanical power (MP) increased progressively and consistently with higher PEEP levels (both *p* < 0.001; F = 646 and F = 274, respectively). From ZEEP to 16 cmH_2_O, Pplat increased by 18.2 cmH_2_O (95% CI 16.9 to 19.5; *p* < 0.001), while MP increased by 13.4 J/min (95% CI 11.1 to 15.7; *p* < 0.001). These findings reflect a marked and consistent increase in both static and dynamic components of respiratory system loading with incremental PEEP. Detailed pairwise comparisons are presented in Figure 1.

### 3.3. PEEP Volume and Strain Analysis

**Figure 2:**
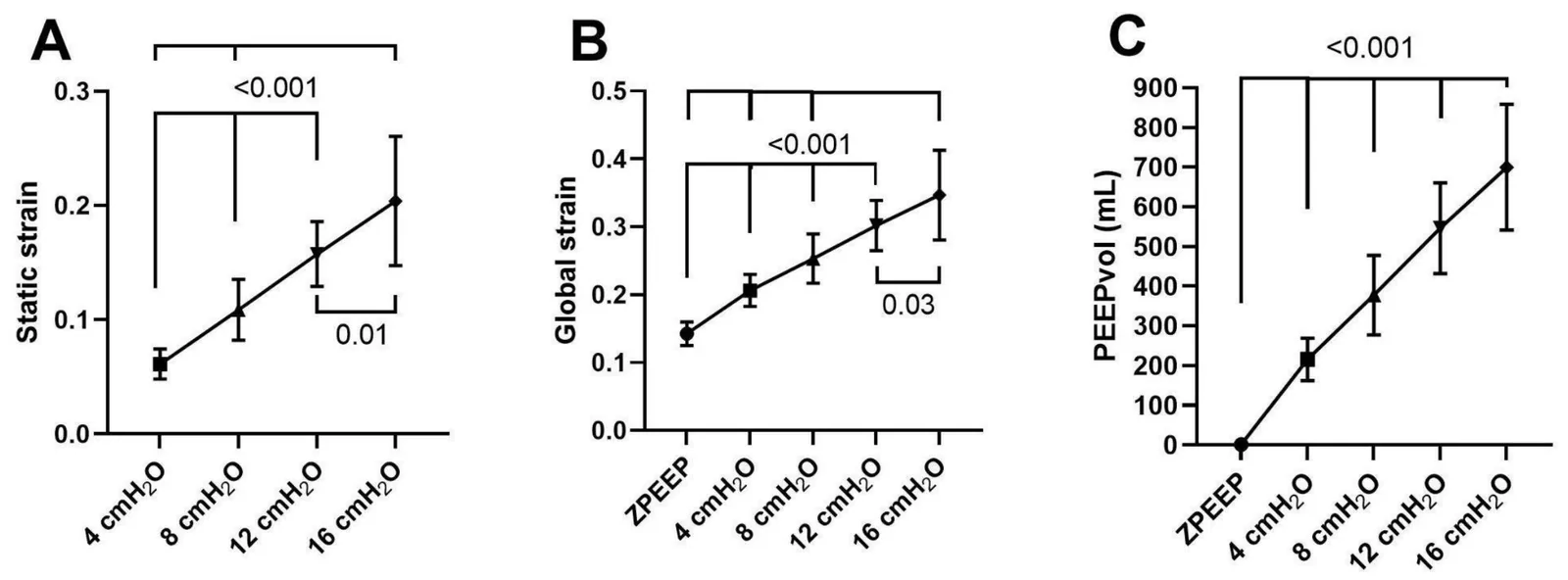
PEEP Volume and Strain Analysis. Data are presented as mean ± standard deviation for each PEEP level. (**A**): Static strain (PEEPvol/FRCt). (**B**): Global strain ((PEEPvol + TV)/FRCt). (**C**): PEEP volume (PEEPVol; PEEP × Cstat). One-way ANOVA with Bonferroni post hoc comparisons was used to assess differences between PEEP levels. Statistical significance was defined as *p* < 0.05. ZEEP was omitted from panel (**A**) because static strain is zero when applied PEEP is zero.

Variations in static strain, global strain, and PEEP-induced lung volume (PEEPVol) across incremental PEEP levels are illustrated in Figure 2. All three parameters increased significantly with escalating PEEP (*p* < 0.001 for all; F = 74.4 for static strain; F = 140 for global strain; and F = 174 for PEEPVol). From ZEEP to 16 cmH_2_O, PEEPVol increased by 699 mL (95% CI 564 to 833; *p* < 0.001), while global strain increased by 0.204 (95% CI 0.156 to 0.252; *p* < 0.001). Static strain also showed consistent stepwise increases across PEEP levels. Given that strain indices are mathematically derived from PEEPVol, these parallel increases should be interpreted considering their inherent structural coupling.

### 3.4. Associations and Predictors of PEEP-Induced Changes

**Figure 3:**
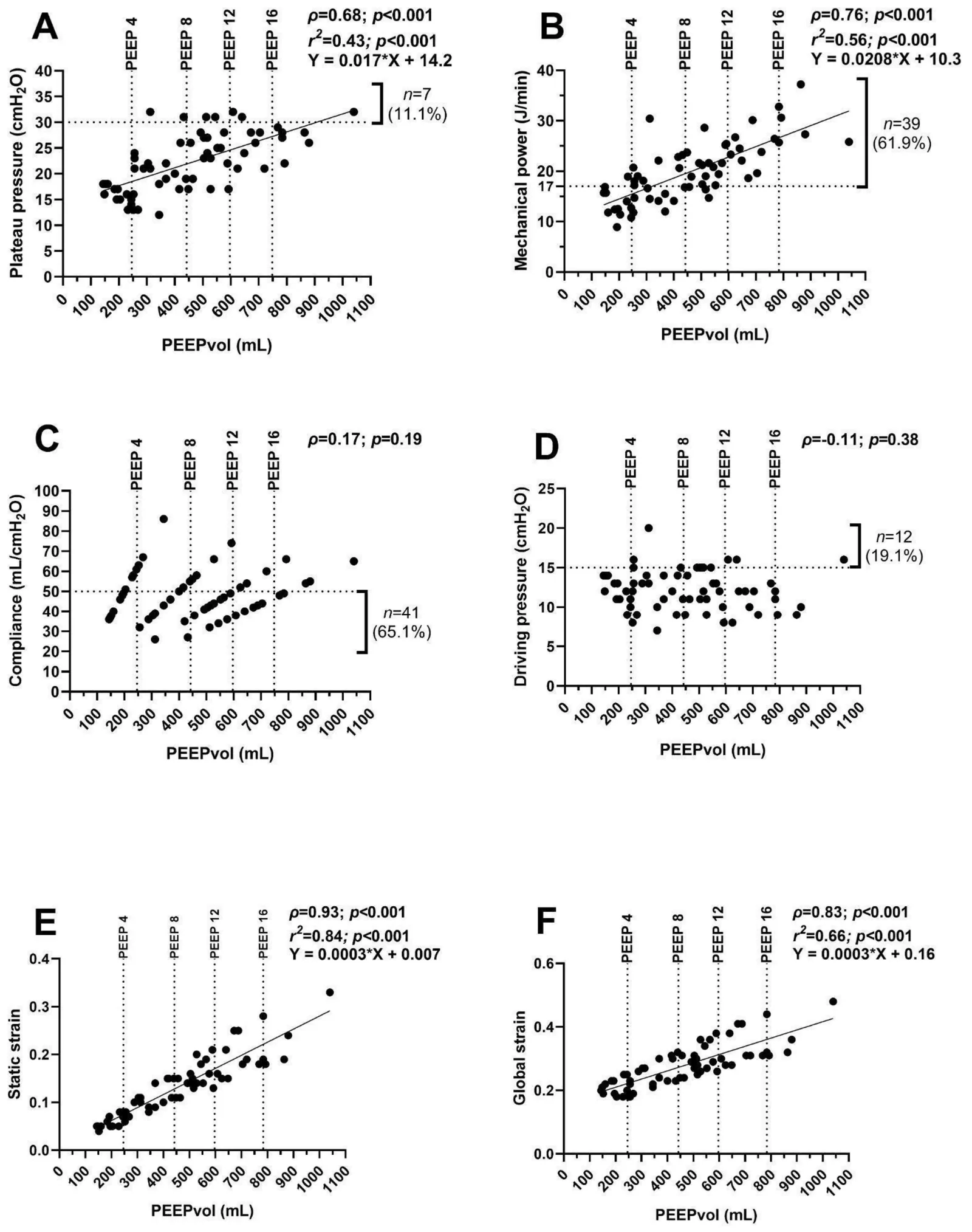
Associations and Predictors of PEEP-Induced Changes. Correlations and linear regressions between dependent variables and PEEP volume (PEEPVol) (*n* = 63). (**A**): Plateau pressure (horizontal dashed line indicates a cutoff of 30 cmH_2_O). (**B**): Mechanical power (horizontal dashed line indicates a cutoff of 17 J/min). (**C**): Static compliance (horizontal dashed line indicates a cutoff of 50 mL/cmH_2_O). (**D**) Driving pressure (horizontal dashed line indicates a cutoff of 15 cmH_2_O). (**E**) Static strain. (**F**) Global strain. The *x*-axis represents PEEPVol values. The vertical dashed line indicates the 75th percentile of the PEEPVol distribution. ρ: Spearman’s rank correlation coefficient. r^2^: Coefficient of determination. Statistical significance was defined as *p* < 0.05.

Correlation analyses between PEEPVol and respiratory mechanics variables are presented in Figure 3. PEEPVol demonstrated moderate-to-strong positive correlations with plateau pressure (ρ = 0.68; 95% CI, 0.51–0.80; *p* < 0.001) and mechanical power (ρ = 0.76; 95% CI, 0.62–0.85; *p* < 0.001). Strong positive correlations were also observed between PEEPVol and static strain (ρ = 0.93; 95% CI, 0.89–0.96; *p* < 0.001) as well as global strain (ρ = 0.83; 95% CI, 0.73–0.90; *p* < 0.001). Conversely, no significant correlations were identified between PEEPVol and static compliance (ρ = 0.17; 95% CI, −0.09 to 0.40; *p* = 0.19) or driving pressure (ρ = −0.11; 95% CI, −0.36 to 0.15; *p* = 0.38) (Figure 3C,D).

Univariable linear regression analyses confirmed significant associations between PEEPVol and plateau pressure (F = 46, *p* < 0.001, r^2^ = 0.43) as well as mechanical power (F = 74, *p* < 0.001, r^2^ = 0.56). Associations with strain indices showed higher coefficients of determination (static strain: r^2^ = 0.84; global strain: r^2^ = 0.66), reflecting the mathematical relationship between these variables and PEEPVol. Given the repeated-measures design, all correlation and regression analyses should be interpreted as exploratory.

To account for the non-independence of repeated observations within patients, additional linear mixed-effects regression models were performed using patient as a random intercept. These additional analyses demonstrated that PEEPVol remained significantly associated with plateau pressure (β = 0.0234; 95% CI 0.0194–0.0270; *p* < 0.001), mechanical power (β = 0.0196; 95% CI 0.0169–0.0224; *p* < 0.001), static strain (β = 0.000294; 95% CI 0.000280–0.000308; *p* < 0.001), and global strain (β = 0.000295; 95% CI 0.000281–0.000309; *p* < 0.001). These findings demonstrated that the observed associations remained robust after accounting for within-subject correlation (Table 2).

### 3.5. Clinical Thresholds Analysis

Across the total pool of 63 measurements, we evaluated the proportion of settings exceeding established safety thresholds: 11.1% (*n* = 7) exceeded a plateau pressure of 30 cmH_2_O, 61.9% (*n* = 39) exhibited mechanical power ≥17 J/min, 65.1% (*n* = 41) showed static compliance ≤50 mL/cmH_2_O, and 19.0% (*n* = 12) presented a driving pressure ≥15 cmH_2_O.

## 4. Discussion

The application of positive end-expiratory pressure (PEEP) inevitably increases end-expiratory lung volume, referred to as PEEP volume (PEEPVol) [16]. PEEPVol is fundamentally determined by the set PEEP level and the mechanical impedance of the respiratory system. In this cohort of mechanically ventilated adults without underlying pulmonary disease, we examined the impact of incremental PEEP titration on PEEPVol and key respiratory mechanics. Although respiratory system compliance (Crs) and driving pressure (ΔP) did not change significantly across PEEP levels, we observed significant increases in static recoil pressure (plateau pressure), PEEPVol, static strain, global strain, and mechanical power.

Furthermore, we identified strong positive correlations between PEEPVol and both static strain (ρ = 0.93; *p* < 0.001) and global strain (ρ = 0.83; *p* < 0.001). Moderate-to-strong correlations were also observed between PEEPVol and plateau pressure (ρ = 0.68; *p* < 0.001) as well as mechanical power (ρ = 0.76; *p* < 0.001). Based on the coefficients of determination, approximately 43% of the variance in plateau pressure and 56% of the variance in mechanical power were attributable to changes in PEEPVol. PEEPVol may help contextualize the mechanical consequences of a given PEEP setting. Exploratory linear regression analysis indicated that for every 100 mL increase in PEEPVol, plateau pressure increased by approximately 1.7 cmH_2_O, and mechanical power by 2.1 J/min.

Although these correlation and linear regression analyses are useful to illustrate the observed physiological relationships, they do not account for the lack of independence among repeated measurements obtained within the same subject. Therefore, we performed additional linear mixed-effects regression models including patient as a random intercept. These mixed-effects analyses demonstrated that PEEPVol remained significantly associated with plateau pressure (β = 0.0234; 95% CI 0.0194–0.0270; *p* < 0.001), mechanical power (β = 0.0196; 95% CI 0.0169–0.0224; *p* < 0.001), static strain (β = 0.000294; 95% CI 0.000280–0.000308; *p* < 0.001), and global strain (β = 0.000295; 95% CI 0.000281–0.000309; *p* < 0.001), indicating that the observed associations remained statistically significant after accounting for within-subject correlation.

### 4.1. PEEPVol, Driving Pressure, and Compliance

These findings corroborate existing literature suggesting that driving pressure has limited and inconsistent prognostic value in unselected patients without ARDS [17,18]. In the largest evaluation to date, Lanspa et al. [17] found that tidal volume—rather than driving pressure—was independently associated with 30-day mortality in non-ARDS patients, while neither driving pressure nor compliance predicted outcome in this population. Similarly, Schmidt et al. [18] reported no independent association between driving pressure and hospital mortality in an unselected non-ARDS cohort; however, when patients were stratified by oxygenation, both driving pressure and compliance became significant predictors of mortality in the hypoxemic subgroup (PF ratio ≤ 300), but not among non-hypoxemic patients. Taken together, these observations suggest that the prognostic value of driving pressure in patients without ARDS is not simply absent but rather emerges once some degree of gas-exchange impairment is present, and that volume-based metrics may carry information that compliance-normalized pressure metrics do not capture in healthier lungs. This is consistent with our own findings: despite the marked increases in PEEPVol and mechanical power across PEEP levels, driving pressure remained essentially unchanged, indicating that ΔP may be insensitive to clinically relevant increases in lung volume and energy transfer in patients without pre-existing lung disease.

### 4.2. PEEPVol and Mechanical Power

Consistent with our observations, a recent study [19] identified mechanical power as an independent risk factor for mortality in neurocritical patients, regardless of the presence of ARDS or obesity. Our results, derived from patients without pre-existing pulmonary disease, suggest that energy transfer and potentially injurious parenchymal deformation may still occur at higher PEEP levels. This aligns with animal models showing an absence of VILI under low global strain, while demonstrating that increasing PEEP at a fixed tidal volume is associated with increased mortality [20]. Notably, a subset of our patients exceeded accepted safety thresholds for plateau pressure (30 cmH_2_O) and mechanical power (17 J/min) even at relatively low PEEP levels (approximately 8 cmH_2_O).

This observation underscores the clinical relevance of these thresholds, as they reflect the energy transmitted per unit time to the lung parenchyma [21] and have been independently associated with increased mortality, prolonged mechanical ventilation, and extended ICU length of stay [22]. This further reinforces the concept that mechanical power includes a significant static component, which argues against the routine application of high PEEP in this subpopulation [9]. Indeed, a meta-analysis of randomized controlled trials reported that mechanical power is independently associated with increased mortality in patients without ARDS [23]. Regarding the quantification of mechanical power, there is an ongoing debate as to whether it should be normalized to predicted body weight or the available surface area for energy absorption [24,25]. Some authors suggest that mechanical power should be adjusted for the mass of pulmonary tissue involved in ventilation, a parameter termed “intensity” [26]. The rationale is that for any given mechanical power, intensity increases as the ventilated lung surface area decreases [27]. While this implies that healthy lungs may possess greater capacity to absorb mechanical energy, excessive PEEP may increase static elastic load without necessarily translating into additional benefit in lungs with low recruitability [28].

### 4.3. PEEPVol, PEEP, and Volume Overdistension

Vieira et al. [29] demonstrated that PEEP can induce both alveolar recruitment and overdistension, depending on the patient’s underlying lung state. In our cohort, the observed increases in plateau pressure, strain, and mechanical power with rising PEEPVol may predominantly reflect inflation of previously aerated lung regions rather than true tissue recruitment. The strong correlations between static/global strain and PEEPVol suggest that increasing lung gas volume via PEEP leads to greater static alveolar stretch and deformation. Similarly, the correlations with plateau pressure and mechanical power indicate that increasing PEEPVol in healthy lungs results in higher distending pressures and increased energy transmission to the parenchyma. We must acknowledge a critical methodological caveat regarding the exceptionally high correlations observed between PEEPVol and both static (*ρ* = 0.93) and global lung strain (*ρ* = 0.83). Because static strain is mathematically defined as PEEPVol/FRCt—where FRCt functions as a fixed, anthropometrically predicted constant for each individual subject—a strong linear association between these variables is structurally mandatory rather than purely biological. This phenomenon, known as mathematical or structural coupling, implies that these specific regression models do not unveil a novel physiological interaction. Instead, they represent an algebraic redundancy inherent to the strain formula itself. Consequently, these findings should be interpreted strictly as a quantification of the physical scaling of delivered volume relative to predicted lung size, rather than an independent pathophysiological correlation. The fact that some subjects surpassed established “safe” thresholds (plateau pressure 30 cmH_2_O; mechanical power 17 J/min) even at low PEEP levels highlights the potential for overdistension, even in the absence of pre-existing lung disease.

The Recruitment-to-Inflation ratio (R/I ratio), proposed by Chen et al. [30] and analyzed extensively by Chatburn et al. [31], seeks to quantify the fraction of volume specifically attributable to true tissue recruitment, normalized for baseline compliance. While PEEPVol in our study appears to primarily reflect the cost of static deformation, the R/I ratio attempts to estimate the benefit regarding alveolar recruitment. Integrating both metrics could provide a more comprehensive clinical assessment of elastic recruitment versus overall alveolar recruitment.

Finally, it is essential to distinguish between gas recruitability—the inflation of already patent alveoli—and true tissue recruitability, which involves the opening of previously collapsed or fluid-filled alveolar units [16,32]. While superimposing pressure-volume (P-V) curves can assess recruitability (where deviation, or hysteresis, between curves indicates recruitment), this methodology requires rigorous interpretation. Given that our cohort lacked pre-existing lung disease, the observed changes in respiratory mechanics primarily reflected gas recruitment rather than tissue recruitment. In patients with pulmonary pathologies, however, PEEP’s effects are more complex, potentially involving simultaneous over-stretching and recruitment [16]. Studies comparing P-V curves and helium dilution techniques for estimating recruitable volume have shown moderate agreement, yet they differ from computed tomography (CT) assessments [33]. This discrepancy likely arises because CT primarily detects the reopening of collapsed alveoli, whereas mechanical methods estimate both recruitability and volume changes in already open areas. Furthermore, methods measuring end-expiratory lung volume (EELV)—such as CT or gas dilution—express recruited volume relative to baseline, whereas P-V curves do not always account for this. Similarly, Stahl et al. [16] questioned the reliability of volume recruitment based on EELV changes, demonstrating that this value may be higher in healthy lungs than in those with ARDS, suggesting that EELV increases may reflect the slow inflation of aerated tissue rather than the recruitment of collapsed alveoli.

### 4.4. Clinical Implications and Future Directions

As this study is hypothesis-generating, its bedside implications should be framed cautiously. Because PEEPVol can be calculated from variables already available at the bedside (applied PEEP and static compliance), it could in principle be tracked in real time during PEEP titration as an adjunct to plateau pressure and mechanical power, flagging patients in whom relatively low airway pressures are nonetheless associated with disproportionate volume and strain increases. Before such use can be recommended, however, PEEPVol needs to be benchmarked against direct measures of overdistension and recruitment (EIT or CT) and tested prospectively in more heterogeneous populations, including patients with ARDS or reduced lung compliance, in whom the volume-pressure relationship differs substantially from that observed in this cohort. If validated, PEEPVol-informed PEEP titration could eventually be incorporated into individualized, mechanics-based ventilation strategies rather than used as a standalone threshold.

### 4.5. Limitations

This study has several limitations. First, the cross-sectional design and limited sample size, combined with the absence of pulmonary pathology, preclude complex statistical analyses to determine the long-term effects of varying PEEPVol levels and limit the generalizability of our findings to patients with ARDS or other severe pulmonary diseases. Second, the absence of electrical impedance tomography (EIT) limited our ability to assess the regional distribution of ventilation or to definitively detect localized areas of overdistension or atelectasis. Third, the lack of esophageal catheters prevented the evaluation of the relative contributions of the chest wall and lung to overall respiratory system elastance, which could have been quantified using the elastance ratio (EL/Ers). Fourth, estimating FRC using predictive equations introduces inherent imprecision. Functional residual capacity was derived from demographic predictive equations rather than direct measurement. Predictive models cannot account for the immediate reduction in FRC triggered by general anesthesia and supine positioning due to alveolar collapse [34]. Consequently, using an unadjusted theoretical FRC value introduces an unquantified baseline bias, potentially underestimating the true lung strain; however, previous studies have demonstrated reasonable correlation between estimated and measured FRC values [14,15]. Finally, we did not account for critical opening pressure, as its prevalence in healthy lungs is negligible compared to that observed in ARDS populations. Conversely, evaluating all patients using the same ventilator, under strictly standardized conditions, and with a unified clinical team represents a key strength of this study. This standardization minimizes variability, particularly given that different flow sensors across various ventilator models may introduce inaccuracies in volume measurement [33,35].

Finally, although the correlation and linear regression analyses are useful for visualizing the observed physiological relationships, they do not account for the repeated-measures nature of the dataset. For this reason, the principal associations were additionally evaluated using linear mixed-effects regression models accounting for repeated measurements within subjects.

## 5. Conclusions

In this secondary analysis of mechanically ventilated adults without pre-existing lung injury, incremental PEEP titration was associated with progressive increases in PEEP-induced lung volume, plateau pressure, strain indices, and mechanical power, despite minimal changes in compliance and driving pressure. These findings suggest that PEEPVol may provide complementary physiological information regarding the mechanical consequences of PEEP titration beyond airway pressure alone. However, in the absence of direct measurements of recruitability or regional overdistension (e.g., electrical impedance tomography, esophageal pressure, or CT imaging), our data cannot establish whether increased PEEPVol reflects harmful overdistension rather than physiological lung inflation. These observations should be considered hypothesis-generating.

## Figures and Tables

**Table 1:** Cohort baseline characteristics.

Variable	Total (*n* 16)
Male	81.25% (*n* 13)
Age (years)	57.06 ± 16.32
Weight (kg)	67.55 ± 8.59
Height (cm)	172.6 ± 8.45
FRCt (mL)	3478 ± 588.9
APACHE II	21.6 ± 4.4
Causes of mechanical ventilation	
Consciousness deterioration	5 (31.25%)
Abdominal surgery	4 (25%)
Cardiovascular surgery	3 (18.75%)
Cardiorespiratory arrest	2 (12.5%)
Head trauma	2 (12.5%)
OT diameter (mm)	8 (8–8)
MV (days)	1.2 ± 0.4
Ventilator Mode (VC-CMV)	100% (*n* 16)
Tidal Volume (mL)	492.5 ± 50.4
Respiratory rate (cycles/min)	18.4 ± 2.1
PEEP (cmH_2_O)	5 (5–7.5)
FiO_2_ (%)	36.3 ± 7.7
SpO_2_	96.3 ± 2.09
Peak Inspiratory Flow (L/min)	60 (60–60)
TV/PBW (mL/kg)	7.27 ± 0.6

Data are expressed as mean and standard deviation or median and interquartile range (IQR 25–75%) according to the data distribution. FRCt: theoretical functional residual capacity; MV: mechanical ventilation; OT: orotracheal tube; VC-CMV: volume control − continuous mandatory ventilation; PEEP: positive end-expiratory pressure; SpO_2_: pulse oximetry; TV: tidal volume; PBW: predicted body weight.

**Table 2:** Linear mixed-effects regression models evaluating the association between PEEPVol and respiratory mechanics. β corresponds to the fixed-effect coefficient for PEEP-induced lung volume (PEEPVol). Models included patient as a random intercept.

Outcome	β (PEEPVol)	95% CI	*p*
Plateau pressure	0.0234	0.0194–0.0270	<0.001
Mechanical power	0.0196	0.0169–0.0224	<0.001
Static strain	0.000294	0.000280–0.000308	<0.001
Global strain	0.000295	0.000281–0.000309	<0.001

## Data Availability

The datasets generated and/or analyzed during the current study are not publicly available due to institutional regulations on restrictions on disclosure of health information but are available from the corresponding author on reasonable request.

## Abbreviations

The following abbreviations are used in this manuscript:

PEEP	Positive end-expiratory pressure
PEEPVol	PEEP-induced lung volume
Cstat	static compliance
Pplat	Plateau pressure
EELV	end-expiratory lung volume
ARDS	Acute respiratory distress syndrome
EL/Ers	Elastance ratio
FRCt	theoretical functional residual capacity
